# Efficacy and feasibility of a 12-week Tai Chi training for the prophylaxis of episodic migraine in Hong Kong Chinese women: A randomized controlled trial

**DOI:** 10.3389/fpubh.2022.1000594

**Published:** 2022-12-13

**Authors:** Yao Jie Xie, Longben Tian, Stanley Sai-Chuen Hui, Jing Qin, Yang Gao, Dexing Zhang, Tongyu Ma, Lorna Kwai Ping Suen, Harry Haoxiang Wang, Zhao-Min Liu, Chun Hao, Lin Yang, Alice Yuen Loke

**Affiliations:** ^1^School of Nursing, Faculty of Health and Social Sciences, The Hong Kong Polytechnic University, Hong Kong, Hong Kong SAR, China; ^2^Research Center for Chinese Medicine Innovation, The Hong Kong Polytechnic University, Hong Kong, Hong Kong SAR, China; ^3^Department of Sports Science and Physical Education, The Chinese University of Hong Kong, Hong Kong, Hong Kong SAR, China; ^4^Department of Sport, Physical Education, and Health, Hong Kong Baptist University, Hong Kong, Hong Kong SAR, China; ^5^JC School of Public Health and Primary Care, The Chinese University of Hong Kong, Hong Kong, Hong Kong SAR, China; ^6^Department of Health Sciences, Franklin Pierce University, Rindge, NH, United States; ^7^School of Nursing, Tung Wah College, Hong Kong, Hong Kong SAR, China; ^8^School of Public Health, Sun Yat-sen University, Guangzhou, China; ^9^College of Medicine and Veterinary Medicine, The University of Edinburgh, Edinburgh, United Kingdom

**Keywords:** migraine, prophylaxis, Tai Chi, Chinese, women, randomized controlled trial

## Abstract

**Background:**

Tai Chi has been broadly applied as alternative treatment for many neurological and psychological disorders. Whereas no study using Tai Chi as prophylactic treatment for migraine. The purpose of this study was to preliminarily examine the efficacy and feasibility of a 12-week Tai Chi training on migraine attack prevention in a sample of Chinese women.

**Methods:**

A two-arm randomized controlled trial was designed. Women aged 18 to 65 years and diagnosed with episodic migraine were randomized to either Tai Chi group (TC group) or the waiting list control group. A modified 33-short form Yang-style Tai Chi training with 1 h per day, 5 days per week for 12 weeks was implemented in the TC group, with a 12-week follow up period. The control group received a “delayed” Tai Chi training at the end of the trial. The primary outcome was the differences in attack frequency between 4 weeks before baseline and at the 9–12 weeks after randomization. The intensity and duration of headache were also measured. The feasibility was evaluated by the maintenance of Tai Chi practice and satisfactory level of the participants toward training.

**Results:**

Eighty-two women were randomized, finally 40 in TC group and 33 in control group were involved in the analysis. On average, women in TC group had 3.0 times (95% CI: −4.0 to −2.0, *P* < 0.01) and 3.6 days (95% CI: −4.7 to −2.5, *P* < 0.01) reduction of migraine attack per month. Compared with the control group, the differences were statistically significant (−3.7 attacks/month, 95% CI: −5.4 to −1.9; and −3.0 migraine days/month, 95% CI: −4.5 to −1.5; both *P* < 0.001). The intensity and duration of headache had 0.6 (95% CI: −1.2 to −0.0, *P* < 0.05) units and 1.2 (IQR: −5.0 to 1.1, *P* < 0.05) hours reduction in TC group, respectively. Most of the participants (69.2%−97.4%) were satisfied with the training. At the end of 24 weeks, on average, the participants maintained 1.5 times of practice per week and 20 min for each practice.

**Conclusion:**

The 12-week Tai Chi training significantly decreased the frequency of migraine attack. It was acceptable and practicable among female migraineurs.

**Clinical trial registration:**

www.ClinicalTrials.gov, identifier: NCT03015753.

## Introduction

Migraine is among the most common primary headache disorders worldwide. According to the Global Burden of Disease Study updated in 2019, migraine caused 4.9% of total years lived with disability (YLDs) in both genders, and took the first place in young women (1, 2). The age-standardized disability-adjusted life years (DALYs) of migraine increased from 22nd highest ranking in 1990 to 14th in 2019 among the 369 diseases and injuries (1, 3). Globally, the prevalence of migraine ranged from 9% to 35% across countries (4). Women was two to three times higher than men (5), the age-standardized prevalence was 18.9% for women and 9.8% for men (6). In Hong Kong, the prevalence ranged from 8.4 to 12.5%, which was as high as hypertension (7, 8). Migraine is usually nonfatal but disabling because repeated migraine attacks are pain and personal suffering, which substantially impair quality of life and increase financial cost (9). The disabled productivity during migraine attack also reduced working hours and working effectiveness (10). Furthermore, evidence showed that frequent attack was highly associated with an elevated risk of developing cardiovascular disease (11–13) and progression of white matter lesions (14), with the consequences of increased neurologic deficits, morbidity and mortality. The prevention of migraine attack is thereby of important public health concern.

The pathogenesis of migraine is believed to be highly complex involving neuronal, inflammatory, and vascular mechanisms. Neural events lead to dilation of blood vessels, which in turn aggravates the pain and results in further nerve activation. Cortical spreading depression (CSD) and brainstem generator are the two concepts of migraine genesis (15). Nonpharmacological treatment plays an important role in the prophylaxis of migraine. Compare with pharmacological approach, nonpharmacological prophylaxis is relatively safer, better tolerated, and associated with improved patient satisfaction (16). As one of the most promising nonpharmacological interventions, exercise is recommended for migraine prophylaxis in recent years (17–19). Potential mechanism links to improvement in neuroinflammatory, neurovascular, neurolimbic, and neuroendocrine processes, and/or psychological and behavioral factors (18, 19). However, exercise itself might also be a potential trigger (20). Improper vigorous exercise could initiate attacks through the pathways of hypocretin changes, lactate accumulation, and systolic blood pressure and cardiac output increases (21). Thus, the type, frequency, and intensity of exercise should be carefully determined for migraine prophylaxis. Tai Chi, a traditional Chinese martial art that has been widely practiced in Chinese population and spread worldwide, is a moderate mind–body exercise that integrates physical and spiritual elements to slowly and gently move *qi* (vital energy) throughout the body. By integrating the movements with deep breathing and mental concentration, mind-body communication is enhanced, allowing a practitioner to achieve a state of harmony between mind and body. Its significant physiological and psychosocial benefits on health outcomes have been well documented in the literature (22). However, its effectiveness among migraineurs remains largely unknown.

There was no published study using Tai Chi as prophylactic treatment for migraine. Nonetheless, Tai Chi has been broadly applied in treatment of mental and psychological disorders. It showed significant beneficial effects on reducing the severity of headache, improving energy expenditure, emotional well-being and mental health for general headache (23). Recent systematic review indicated that Tai Chi can relieve stress, improve sleep quality, alleviate fatigue level, and accordingly promote health-related quality of life and wellbeing (24, 25). As stress, sleep disturbances and fatigue are typical migraine triggers (26), Tai Chi may prevent migraine attacks through this indirect way. Thus, we believe that Tai Chi holds therapeutic potential in migraine prophylaxis. We thereby designed a randomized controlled trial, using Yang-style Tai Chi training as intervention, to preliminarily examine its efficacy in migraine prophylaxis, and test the feasibility of practicing Tai Chi in the study population. We hypothesized that the 12-week Yang style Tai Chi training could significantly decrease the frequency of migraine attacks among Hong Kong Chinese women with episodic migraine.

## Methods

### Study design and setting

This study was a two-arm individual-level randomized controlled trial (RCT). Participants in the intervention group received a 12-week modified short-form Yang-style Tai Chi training with additional 12-week follow-up, and participants in the waiting list control group just kept their usual exercise and lifestyles for 24 weeks and then took the Tai Chi training. The study was implemented in a University in Hong Kong.

### Participants

#### Eligibility criteria for participants

Hong Kong Chinese women who had a clinical diagnosis of episodic migraine (≤ 15 migraine days per month) was the study population. The inclusion criteria were: (1) female, aged 18–65 years; (2) have a clinical diagnosis of episodic migraine with or without aura according to the third edition of the International Classification of Headache Disorders (ICHD-III beta version) (27) at least 2 months prior to enrollment; (3) more than two migraine attacks in 1 month; (4) at least one of the following migraine characteristics is met: nausea, vomiting, photophobia, or phonophobia; (5) able to undertake designated level of Tai Chi exercise; (6) live in Hong Kong, can read and speak Cantonese or Putonghua; (7) give written informed consent. The exclusion criteria were those with: (1) severe migraine attacks with disabilities that preclude moderate intensity physical activity; (2) secondary headache and other neurological disease; (3) more than 5 days of non-migrainous headache per month; (4) experience with Tai Chi practice after diagnosis of migraine; (5) regular performance of Tai Chi or other mind-body exercises (yoga, biofeedback, meditation, etc.); (6) undergoing other alternative therapeutic treatments during recruitment period, or received other alternative therapeutic treatments in the past 12 weeks; (7) pregnancy, lactation period, or currently using contraceptives; (8) use of pharmacological prophylactic treatment for migraine in the past 12 weeks; (9) drug use, take antipsychotic or antidepressant drugs, or take analgesics for other chronic pain more than 3 days a month in the past 12 weeks; and (10) epilepsy, or have a psychiatric disease.

#### Sample size calculation

Since there was no previous intervention study using Tai Chi as migraine prophylaxis, by referring to other complementary treatments, like aerobic exercise (19) and acupuncture (28), we expected 1 attack reduction per month after the Tai Chi intervention. Thus, a sample of 30 in each group was needed to achieve 85% power to detect a difference of 1.0 time (SD: 1.6) reduction in attack, with the significant level of 0.05 for two-sides. By consideration of 10% drop out rate (29), additional five subjects were recruited for each group.

#### Recruitment

Recruitment was done *via* mass media and internet, including university's internal email system, school's alumni system, WhatsApp, and fliers and posters disseminated in the communities and clinics. Information with details of the RCT and an enquiry phone number and email address was displayed in the posters. During the initial contact, the trained research assistants (RAs) briefed prospective participants about the purpose and logistics of the study, evaluated their initial eligibility by a screening form. Those met the basic criteria were involved in 4 weeks observation subsequently, which required the prospective participants to record the migraine attacks through a migraine diary. The migraine diary is a commonly used tool to record frequency, intensity, duration, and relevant medication of migraine attacks for migraineurs. RAs collected the diaries and consulted the collaborative neurological physician at the University Health Service Center, the latter made the final diagnosis according to the ICHD-III beta version criteria (27). The subjects who met all inclusion and exclusion criteria were invited as eligible participants. Before the study, participants were informed of relevant precautions. If unbearable headache occurred during the trial, they could take acute medication for migraine to relieve the headache (e.g., triptans) as advised by their doctors. They were required to record the name and dosage of medication on the migraine diary. The acute medication was only for symptoms alleviation, it would not influence the prophylaxis effect of Tai Chi on the migraine attacks.

### Randomization, blinding, and concealment

A computer random number generator was used to generate the random allocation sequence. Eligible participants were randomly assigned in a 1:1 ratio to one of the two groups: (1) a Tai Chi training group; or (2) a waiting list control group (“control”). Randomization was carried out using a permuted block algorithm with blocks of size 4. To ensure allocation concealment, the RAs assigned a code to each participant and generated several random allocation sequences for each block of size 4. The principal investigator then chose one from each group of generated allocation sequences without knowing the participant identity. Investigators were concealed about the random allocation until the assignments had been made. The RAs who performed the outcome measurements were blinded to the treatment group assignment.

### Interventions

#### Tai Chi training

The 12-week Tai Chi training was prescribed with three 1-h instructor-led sessions and two 1-h self-practice sessions per week. Qualified Tai Chi instructors were recruited from the Gentle and Tranquil Tai Chi Chuan Association, to teach the participants a modified 33-short form Yang-style Tai Chi Chuan, which is the most popular and widely practiced form of Tai Chi in the world. This form is adapted from original 32-short form Yang-style Tai Chi Chuan by including the last form “closing”. It is typically done with slow, steady movements, which is a practical entry point for many beginners. The recruited Tai Chi instructors attended a training session before the commencement of the intervention, to ensure that they agreed on the exact procedure of the Tai Chi intervention protocol and would adhere to the protocol throughout the study.

Each 1-hour training session consisted of 10 min brief warm-up stretching movements followed by 45-min standard Tai Chi routine activities, and 5 min of cool-down stretching. Every instructor-led training session had 15–18 participants, which were performed at an open space with relatively less pedestrians in the University. Also, handouts about the Tai Chi movements and lesson schedule were distributed to the participants to facilitate their learning and practice. Two parallel Tai Chi classes were arranged for the participants. Class A was scheduled on Monday, Wednesday, and Friday; class B was set on Tuesday, Thursday, and Saturday. Both classes followed the same intervention protocol (Supplementary material). Participants could choose one of the two classes according to their available date. To ensure the fidelity of the intervention, the Tai Chi instructors were required to follow the intervention protocol to deliver the Tai Chi training. The RAs monitored all the instructor-led Tai Chi sessions on spots. With the permission of the Tai Chi instructors, the RAs videoed their movements and sent the videos to the participants right after each instructor-led session, to facilitate participants recalling and practicing the Tai Chi forms. Participants were asked to record the date, time, duration, and Tai Chi forms of their self-practices as well as daily physical activities in an exercise log. They were also encouraged to video the self-practices and share the videos in a WhatsApp group that involved all the participants from the intervention group. RAs reviewed exercise logs and WhatsApp group at least twice a week to check whether the participants followed the intervention protocol. If not, RAs would contact them and discussed with them the barriers and challenges toward the Tai Chi training, and encouraged them to follow the intervention protocol.

#### Waiting list control

Participants randomly assigned to the control group were asked to maintain their usual exercise and lifestyles for 24 weeks. At the trial end, they were offered Tai Chi training similar as Tai Chi group. The arrangement of waitlist intervention was intended to provide the participants opportunity for Tai Chi training, and to reduce dropout rate. A delayed Tai Chi training for them might encourage them to stay in the study. Both Tai Chi group and control group participants received a HK$100 supermarket coupon at baseline, those in control group received another HK$100 supermarket coupon at 12 weeks, as an additional incentive to encourage them to keep participation until the end of the study.

### Outcome measures

#### Primary outcome

The primary outcome was the changes in frequency of migraine attack. It was calculated as: (1) the difference in the number of attacks per month between 4 weeks before randomization and weeks 9–12/21–24 after randomization; and (2) the difference of migraine days per month between 4 weeks before randomization and weeks 9–12/21–24 after the randomization. We considered the end of the 12th week as the primary time point. The monthly frequency of migraine attack was defined as the number of attacks per month. The monthly migraine days were defined as the total days that the participant suffered the migraine attacks per month. Participants firstly self-recorded each migraine attacks by migraine diary 4 weeks before the baseline, and then self-recorded from once the intervention commenced until the end of the trial.

#### Secondary outcomes

##### Intensity and duration of headache

The migraine diary was used to record these variables. Intensity of headache was measured by a Visual Analog Scale (30) integrated into the migraine diary. Duration of headache attack was defined as the time of onset of headache to the time of headache disappeared, which was recorded to the nearest 0.1 hour. Participants were asked to record this information soon after experiencing the headache attack. The changes of intensity and duration from the baseline to 12 and 24 weeks were then calculated.

##### The proportion of responders

This was defined as the proportion of patients with at least a 50% reduction of the number of attacks per month (28). The 50% reduction of attacks at 12 and 24 weeks in each group were calculated respectively.

##### Feasibility of the Tai Chi training

Feasibility was defined as how successful the Tai Chi intervention is implemented. It was evaluated by the duration of recruitment, retention rate in Tai Chi training, and maintenance of the Tai Chi self-practice. The RAs also monitored and recorded the adverse effects from the participants. We defined an instructor-led session attendance rate of < 10% as invalid attendance. The participants were encouraged to follow the Tai Chi protocol to practice for a certain amount of time during each self-practice session, which was not < 20 min (31). The maitemnance of Tai Chi self-practice was determined in two aspects: (1) what percentage of the participants in the intervention group performed Tai Chi exercise for at least 4 weeks in the 12-week follow up period; and (2) how many weeks in the 12-week follow up period the participants in the intervention group practiced Tai Chi at least once per week (32).

#### Covariates

At baseline, a structured interview was conducted to collect information on the socio-demographic characteristics, medical history, physical activity, dietary intake, lifestyle factors (drinking and smoking), reproductive information, and family history of migraine. Anthropometric measurements, including weight, height, waist and hip circumference, and percent body fat, were taken, with participants wearing light clothing and following standard protocols. Weight was measured to the nearest 0.1 kg and height to the nearest 0.1 cm using a calibrated scale with a height bar. Waist and hip circumference were measured using a tape measure to the nearest 0.1 cm. Body fat percentage was measured by bioelectrical impedance analysis (Tanita, BC 581, Japan). Body mass index [BMI, weight (kg)/height (m^2^)] and waist-to-hip ratio (waist circumference/hip circumference) were calculated. Furthermore, the typical migraine triggers including fatigue, stress level, and sleep quality were assessed by the numeric rating scale-fatigue, the 14-item Perceived Stress Scale, and the Pittsburgh Sleep Quality Index, respectively (33). All above measurements at baseline were conducted again at the 12th and the 24th week.

### Statistical analysis

Missing values were handled by multiple imputations. Mean and standard deviation (SD) or median and interquartile range were used to describe continuous variables where appropriate. Categorical variables were presented as numbers and percentage (%). Independent *t*-test, Mann-Whitney *U-*test, Pearson chi-square test or Fisher's exact test, were conducted to compare the differences between groups for normal distributed, skewed, and categorical data, respectively. The 12-week and 24-week changes for each outcome variable in each group were calculated firstly. Then the paired *t*-test, Wilcoxon signed-rank test, and McNemar test were used for within group comparison for normal distributed, skewed, and categorical data, respectively. To finally test intervention effects between groups, differences in changes were compared across the two arms using repeated analyses of covariance (ANCOVA), with adjustment of baseline characteristics; the time × group interaction effects across baseline, 12-week and 24-week were examined subsequently. Both per-protocol analysis and Intention to Treat (ITT) analysis were adopted according to the CONSORT guidelines (34). Statistic software SPSS 23.0 (SPSS Institute) was used for analysis. All statistical tests were two-sided and a *p*-value < 0.05 was considered statistically significant.

## Results

From 2016 to 2017, 189 women who indicated initial interests in participating were enrolled. After screening of eligibility, 80 women were excluded according to the inclusion and exclusion criteria. The remaining 109 subjects were made appointment for baseline measurement. While 26 of them canceled due to the time conflict, and one woman did not attend because of health problem. A total of 82 participants completed the baselines measurement. They were randomly allocated to Tai Chi group (*n* = 42) and control group (*n* = 40). After the allocation, two participants in the Tai Chi group and seven participants in the control group withdrew immediately. The former two participants withdrew due to time conflict. Those seven participants in the control group withdrew because they had high expectations to be selected to the Tai Chi group. As result, a total of 40 participants started the Tai Chi training in the intervention group, and 33 participants remained in the control group, who were considered as valid participants.

**Figure 1:**
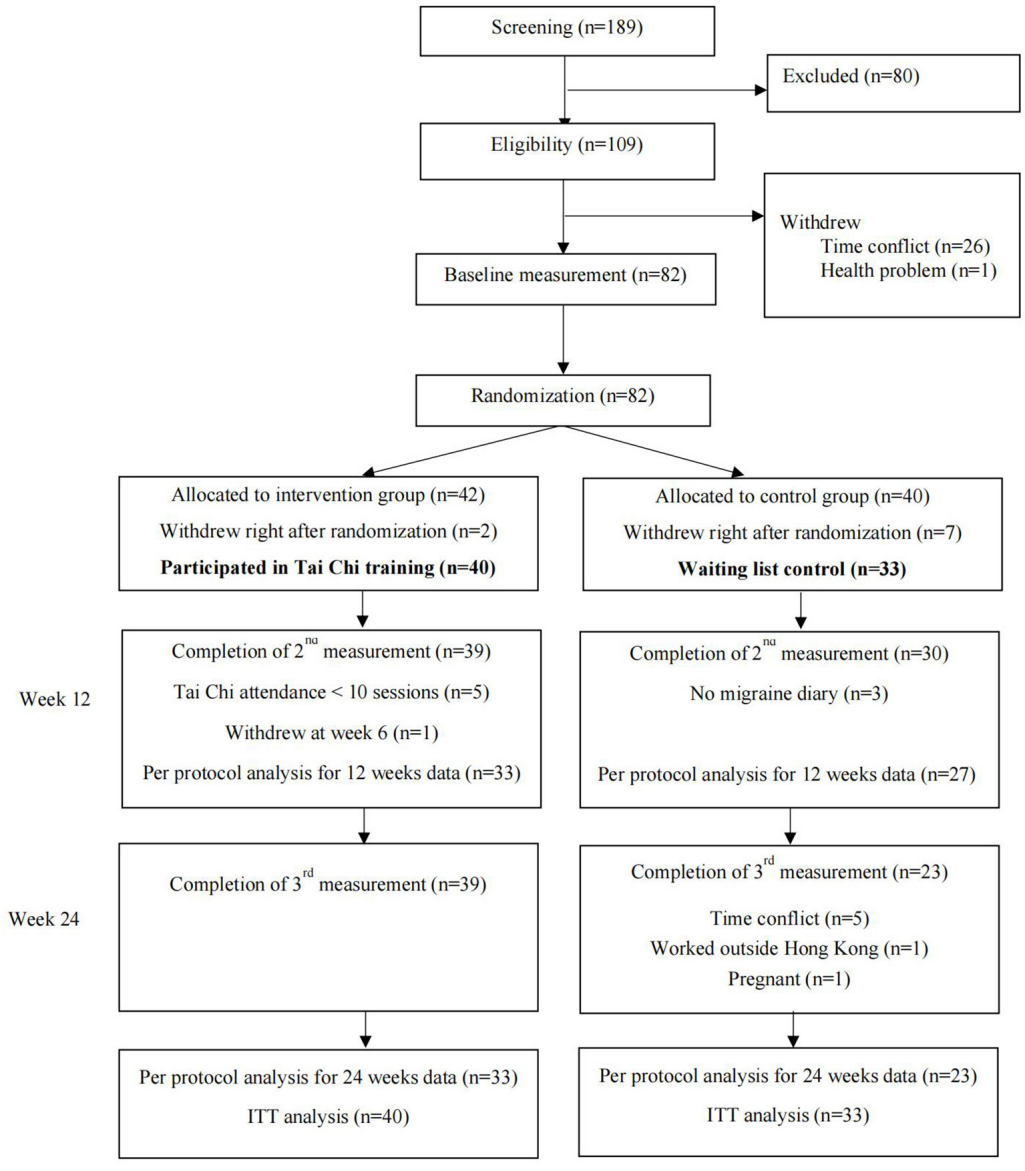
Flow diagram of the study procedure.

After 12 weeks intervention, 39 participants in the Tai Chi group and 30 participants in the control group completed the 2nd round data collection. During the 12 weeks follow up period, all participants in the Tai Chi group remained in the study and completed the 3rd round data collection, while seven participants in the control group dropped out due to personal reasons. The five participants who attended < 10 Tai Chi sessions were not considered as valid attendance and were excluded from the per protocol analysis. The whole study flow was shown in Figure 1, indicating the exact number of participants in each period, from the enrollment to the data analysis. The retention rates at the 12th and 24th week were both 98% (39/40) for the Tai Chi group, and 82% (27/33) and 70% (23/33) for the control group, respectively. No significant differences were observed in the baseline characteristics between participants who completed the entire study and those who were lost to follow-up (all *P* > 0.05).

### Basic characteristics of the participants

The basic characteristics of the 73 participants were shown in Table 1. The average age was 50.9 ± 10.2 years and 47.1 ± 11.8 years in the Tai Chi group and the control group, respectively. More than half of the participants were employed (58.9%), with a monthly family income between HK$ 20,000 and HK$ 80,000 (64.4%). Most of the participants (95.9%) had secondary or above education level, and the majority of the participants were married (63.0%). The mean BMI and body fat percentage was 22.7 ± 3.5 kg/m^2^ and 32.1 ± 5.4% for the intervention group, and 23.6 ± 3.8 kg/m^2^ and 33.1 ± 4.3% for the control group. Although participants in the control group had higher values of BMI, weight, body fat percentage, and waist circumference when compared with the Tai Chi group, all these anthropometrical values showed no statistically significant difference between two groups (all *P* > 0.05). Also, no difference was observed in terms of fatigue, stress level, and sleep quality (33). The only difference was observed in the drinking, which participants in the control group had higher proportion of drinking than those in the Tai Chi group (*P* < 0.05).

**Table 1:** Basic characteristics of the participants ^a^.

	**Intervention**	**Control**	***p* ^b^**
	**(*n =* 40)**	**(*n =* 33)**	
Age, year(s)	50.9 (10.2)	47.1 (11.8)	0.151
Height, cm	156.2 (6.6)	157.0 (5.4)	0.558
Weight, kg	55.5 (10.0)	58.2 (9.5)	0.247
BMI, kg/m^2^	22.7 (3.5)	23.6 (3.8)	0.276
Overweight/obesity ^#^,^†^	19 (47.5)	16 (48.5)	0.933
Waist circumference, cm	77.5 (9.3)	81.1 (10.8)	0.134
Central obesity ^#^,^†^	16 (40.0)	15 (45.5)	0.639
Hip circumference, cm	94.5 (6.5)	96.3 (8.3)	0.305
Waist-hip ratio	0.8 (0.1)	0.8 (0.1)	0.169
Body fat, %	32.1 (5.4)	33.1 (4.3)	0.383
Hypertension ^#^	2 (5.0)	6 (18.2)	0.13
High Cholesterol ^#^	8 (20.0)	3 (9.1)	0.325
**Physical activity category (by IPAQ)** ^‡^		0.313
Low	6 (15.0)	2 (6.1)	
Moderate	29 (72.5)	26 (78.8)	
High	3 (7.5)	5 (15.2)	
Drinking ^#^	21 (52.5)	25 (75.8)	0.041
Nutritional supplement intake ^#^	10 (25.0)	12 (36.4)	0.292
Medication taken for migraine ^#^	8 (20.0)	8 (24.2)	0.663
Pain relief medication taken ^#^	25 (62.5)	25 (75.8)	0.225
Menopause ^#^	23 (57.5)	13 (39.4)	0.164
Family history of migraine ^#^	11 (27.5)	14 (42.4)	0.181
**Marital status**			0.249
Single	14 (35.0)	6 (18.2)	
Married/cohabitating	22 (55.0)	24 (72.7)	
Divorced/separated/widowed	4 (10.0)	3 (9.1)	
**Education**			0.716
Primary or below	2 (5.0)	1 (3.0)	
Secondary or matriculation	21 (52.5)	15 (45.5)	
Tertiary or above	17 (42.5)	17 (51.5)	
**Occupation**			0.221
Employed	21 (52.5)	22 (66.7)	
Not employed / Retired	19 (47.5)	11 (33.3)	
**Monthly family income** ^‡^			0.538
< $20,000	13 (32.5)	6 (18.2)	
$20,000-$39,999	15 (37.5)	15 (45.5)	
$40,000-$79,999	9 (22.5)	8 (24.2)	
≥$80,000	2 (5.0)	3 (9.1)	

^a^Values reported as mean (SD), n (%) or median (interquartile range) for each group where appropriate. ^b^p-values generated from Pearson chi-square test, Fisher's exact test, independent t-test or Mann-Whitney U-test where appropriate. ^#^Variables were showed the number and percentage that counted “Yes” for each group.^†^Overweight/obesity was defined as BMI≥23.0 kg/m^2^, central obesity was defined as waist circumference≥ 80.0 cm.^‡^There were missing data in variables “Physical activity category” (n = 2) and “Monthly family income” (n = 2).

### Migraine features at baseline

At baseline, the average frequency of migraine attack was 6.3 times/month both in the Tai Chi group and control group. Participants experienced 7.4 ± 3.6 and 8.4 ± 6.5 migraine days per month in Tai Chi and control groups, respectively. The intensity of headache was moderate (4.4 in Tai Chi group and 4.5 in control group, 10 as the most severe status). The median attack duration was 6.7 (Interquartile range, IQR: 3.9–11.9) hours in Tai Chi group and 10.3 (IQR: 3.7–22.7) hours in control group. All these migraine features were not significantly different between two groups (all *P* > 0.05).

### Reduction of frequency, intensity, and duration of migraine attack after Tai Chi training

Table 2 shows the changes in outcomes from the baseline to the 12 and 24 weeks with the comparisons within and between groups. Table 3 shows the between-group differences by the time × group interaction effects with adjustment of baseline characteristics. According to the ITT analysis, at 12 weeks, we observed a significant decrease of 3.0 (95% CI: −4.0 to −2.0) migraine attacks and 3.6 (95% CI: −4.7 to −2.5) migraine days within 1 month in the Tai Chi group (all *P* < 0.01), whereas the control group did not show any significant changes (*P* > 0.05). Compared with baseline, the significant decrease in frequency of migraine attack was also observed at 24 weeks, with reduction of 2.6 attacks and 3.4 days of migraine (all *P* < 0.01) within one month. A slight alleviation of headache intensity was found in the Tai Chi group (−0.6, *P* < 0.05), and the duration of headache was shortened of 1.2 h and 1.8 h at 12 and 24 weeks, respectively. However, compared with the control group, the intensity and duration had no statistically significant difference (all *P* > 0.05). Regarding the proportion of responders, 52.5% participants in the Tai Chi group had 50% reduction of attacks at 12 weeks, this proportion slightly increased to 55% at 24 weeks. In control group, the proportion of 50% reduction of attacks was only 12.1% at 12 weeks and 27.3% at 24 weeks (all *P* < 0.05).

**Table 2:** Changes of migraine features from baseline to 12-week and 24-week.

	**ITT**			**Per protocol** ^**c**^		
**Migraine features^a^**	**Intervention**	**Control**	***p* ^b^**	**Intervention**	**Control**	***p* ^b^**
	**(*n =* 40)**	**(*n =* 33)**				
**Frequency, times of attack/month**						
Baseline	6.3 (3.3)	6.3 (5.9)	0.998			
12-week	3.3 (2.7)	7.0 (7.1)	0.008	3.2 (2.9)	5.8 (5.2)	0.016
24-week	3.7 (4.0)	5.7 (6.3)	0.107	3.6 (4.2)	4.7 (3.6)	0.311
Mean change from baseline to 12-week	−3.0 (−4.0 to −2.0)**	0.7 (−0.9 to 2.2)	< 0.001	−3.1 (−4.2 to −2.0)**	0.7 (−1.0 to 2.4)	< 0.001
Mean change from baseline to 24-week	−2.6 (−3.8 to −1.4)**	−0.6 (−1.8 to 0.5)	0.017	−2.6 (−3.9 to −1.3)**	−0.6 (−2.1 to 1.0)	0.040
50% reduction of attacks at 12-week	21 (52.5)	4 (12.1)	< 0.001	19 (57.6)	4 (13.3)	0.001
50% reduction of attacks at 24-week	22 (55.0)	9 (27.3)	0.017	19 (57.6)	7 (30.4)	0.045
**Number of days with migraine**						
Baseline	7.4 (3.6)	8.4 (6.5)	0.398			
12-week	3.8 (3.3)	7.9 (7.0)	0.004	3.6 (3.5)	6.8 (5.2)	0.005
24-week	4.0 (4.2)	6.5 (6.2)	0.04	3.9 (4.5)	5.5 (3.5)	0.162
Mean change from baseline to 12-week	−3.6 (−4.7 to −2.5)**	−0.5 (−1.6 to 0.5)	0.001	−3.8 (−4.9 to −2.6)**	−0.6 (−17 to 0.6)	< 0.001
Mean change from baseline to 24-week	−3.4 (−4.6 to −2.2)**	−1.9 (−3.3 to −0.5)*	0.136	−3.4 (−4.6 to −2.2)**	−2.2 (−4.0 to −0.4)*	0.195
**Headache intensity, VAS score (0-10)**						
Baseline	4.5 (1.6)	4.4 (1.8)	0.812			
12-week	3.9 (1.7)	4.5 (2.2)	0.209	3.8 (1.8)	4.5 (2.3)	0.192
24-week	3.9 (2.1)	4.4 (2.2)	0.369	3.7 (2.0)	4.1 (1.9)	0.566
Mean change from baseline to 12-week	−0.6 (−1.2 to −0.0)*	0.1 (−0.6 to 0.7)	0.072	−0.6 (−1.3 to 0.1)	0.1 (−0.6 to 0.8)	0.154
Mean change from baseline to 24-week	−0.6 (−1.2 to −0.0)*	−0.1 (−0.8 to 0.6)	0.138	−0.7 (−1.3 to −0.1)*	−0.4 (−1.0 to 0.2)	0.496
**Headache attack duration, hr (s)**						
Baseline	6.7 (3.9 to 11.9)	10.3 (3.7 to 22.7)	0.335			
12-week	5.0 (2.0 to 7.8)	8.0 (3.5 to 14.3)	0.079	4.8 (1.7 to 7.6)	8.8 (3.6 to 15.1)	0.040
24-week	4.9 (2.0 to 9.4)	8.0 (3.6 to 18.9)	0.125	4.6 (2.2 to 9.5)	8.0 (3.8 to 17.4)	0.214
Change from baseline to 12-week	−1.2 (−5.0 to 1.1)*	0.0 (−3.8 to 2.3)	0.668	−1.4 (−6.0 to 1.0)*	0.3 (−4.1 to 2.9)	0.743
Change from baseline to 24-week	−1.8 (−4.5 to 1.4)*	0.0 (−4.0 to 2.8)	0.889	−1.7 (−5.4 to 1.2)	−0.5 (−4.4 to 2.5)	0.648

^a^Values were presented as mean (SD), median (Interquartile range) and n (%) for 12-week and 24-week measurement; mean (95% CI), median (Interquartile range) and % for change from baseline respectively. ^b^p-values generated from Pearson chi-square test, Fisher's exact test, independent t-test and Mann-Whitney U-test where appropriate; univariate ANCOVA was used to compare the mean change difference between groups; variables with significant different between groups at baseline were adjusted as covariates. ^c^Per protocol at 12-week: intervention (n = 33), control (n = 27); at 24-week: intervention (n = 33), control (n = 23). ^*^p < 0.05 generated from within group comparison by paired t-test, Wilcoxon signed-rank test and McNemar test where appropriate. ^**^p < 0.01 generated from within group comparison by paired t-test and McNemar test where appropriate.

**Table 3:** Between-group differences of migraine features at 12-week and 24-week.

	**Between group difference**		**Between group difference**		
	**at 12-week**		**at 24-week**		
**Migraine feature^a^**	**Intervention vs. Control**	***p* ^b^**	**Intervention vs. Control**	***p* ^b^**	***p* ^c^**
**Frequency**					
ITT	−3.7 (−5.4 to −1.9)	< 0.001	−2.0 (−3.7 to −0.3)	0.017	0.001
Per protocol^d^	−3.8 (−5.8 to −1.9)	< 0.001	−2.0 (−4.0 to −0.1)	0.040	0.002
**Number of days with migraine**					
ITT	−3.0 (−4.5to −1.5)	0.001	−1.5 (−3.4 to 0.3)	0.136	0.003
Per protocol	−3.2 (−4.7 to −1.6)	< 0.001	−1.2 (−3.2 to 0.8)	0.195	0.001
**Intensity**					
ITT	−0.7 (−1.5 to 0.2)	0.072	−0.5 (−1.5 to 0.4)	0.139	0.169
Per protocol	−0.7 (−1.6 to 0.3)	0.154	−0.3 (−1.2 to 0.6)	0.496	0.637
**Duration**					
ITT	−0.3 (−5.7 to 5.1)	0.668	−1.5 (−7.2 to 4.2)	0.894	0.747
Per protocol	−1.1 (−7.0 to 4.7)	0.743	−0.9 (−8.1 to 6.3)	0.648	0.759

^a^Values were presented as mean (95% CI) for mean change between groups. ^b^ p-values were calculated for the time × group interaction effects from baseline to 12-week or from baseline to 24-week between groups by repeated ANCOVA; variables with significantly different between groups at baseline were adjusted as covariates. ^c^p-values were calculated for the time × group interaction effects across baseline, 12-week and 24-week between groups by repeated ANCOVA; variables with significantly different between groups at baseline were adjusted as covariates. ^d^Per protocol at 12-week: intervention (n = 33), control (n = 27); at 24-week: intervention (n = 33), control (n = 23).

Compared with control, at 12 weeks, the between-group differences of attacks/month and migraine days/month was−3.7 (95% CI: −5.4 to −1.9) attacks and −3.0 (95% CI: −4.5 to −1.5) days, respectively (both *P* < 0.001). A slightly greater reduction was observed at 24 weeks (Table 3). No significant between-group difference was observed in terms of intensity and duration of headache (all *P* > 0.05) (Table 3). Similar findings were observed by the per protocol analysis (Tables 2, 3).

### Satisfactory level, maintenance, and adverse effects of the Tai Chi training

**Figure 2:**
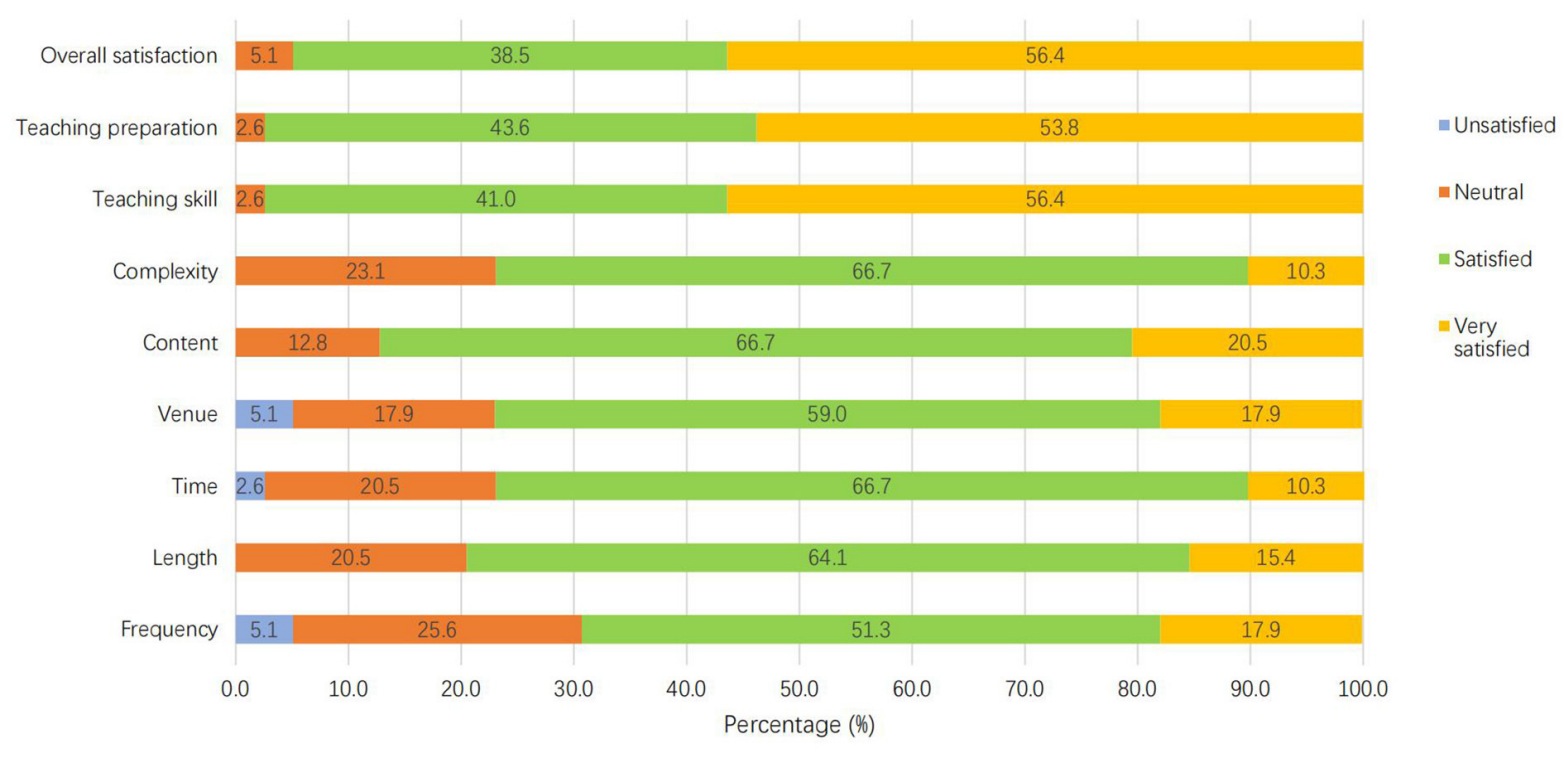
Satisfactory level of the Tai Chi training.

Table 4 shows the participants' satisfactory level in terms of Tai Chi training frequency, length, time, venue, content, complexity, as well as Tai Chi instructors' teaching skill, teaching preparation, and the overall satisfaction level. The great majority of them were satisfied with the training programme. The percentage of those who selected “Satisfied” and “Very satisfied” ranged from 69.2% (for training frequency) to 97.4% (for teaching skill and teaching preparation; Figure 2). The most unsatisfied aspects were the frequency of the training and the venue of practice. Some participants indicated that 5 times training per week was too many, and an indoor practice room was better for practice because it was more private, quieter and cooler.

**Table 4:** Satisfactory level of Tai Chi training (*n* = 39).

	**Unsatisfied (%)**	**Neutral (%)**	**Satisfied (%)**	**Very satisfied (%)**	**Mean (SD)**
Frequency	5.1	25.6	51.3	17.9	3.8 (0.8)
Length	0.0	20.5	64.1	15.4	4.0 (0.6)
Time	2.6	20.5	66.7	10.3	3.9 (0.6)
Venue	5.1	17.9	59.0	17.9	3.9 (0.8)
Content	0.0	12.8	66.7	20.5	4.1 (0.6)
Complexity	0.0	23.1	66.7	10.3	3.9 (0.6)
Teaching skill	0.0	2.6	41.0	56.4	4.5 (0.6)
Teaching preparation	0.0	2.6	43.6	53.8	4.5 (0.6)
Overall satisfaction	0.0	5.1	38.5	56.4	4.5 (0.6)

**Figure 3:**
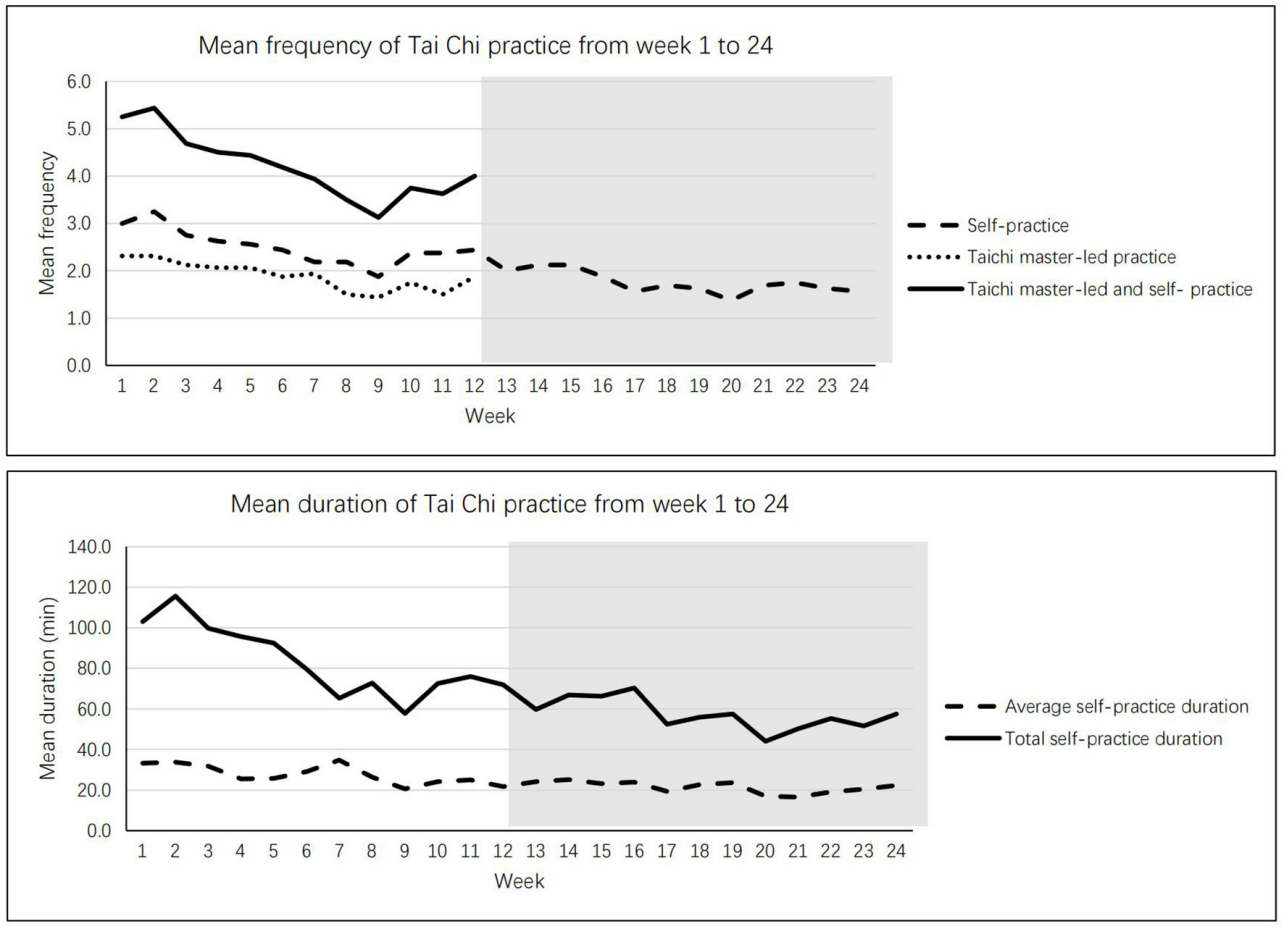
Maintenance of Tai Chi practice from week 1 to week 24.

Regarding maintenance of the Tai Chi practice, Figure 3 shows the times of Tai Chi practice per week and the length of practice for 24 weeks. At the end of the 24 weeks, on average, the participants kept 1.5 times of practice per week and 20 min for each practice. As times went by, the frequency and duration of practice slightly decreased. But all participants in the intervention group kept the Tai Chi practice in the 12-week follow up period, and they practiced at least one time per week during that period. For the adverse effects, some participants reported joint pain (33.8%), muscle pain (33.3%), slight sprain (10.3%), and dizziness (5.1%). But all of them indicated tolerable for these symptoms, no serious case was founded.

## Discussion

To our knowledge, this is the first experimental study using Tai Chi as prophylactic treatment for migraine attack in women. The findings from this study demonstrated that the 12-week Tai Chi training had significant effects on reducing the frequency of migraine attack and the number of migraine days. It could also slightly alleviate headache intensity and shorten the duration of headache after intervention. Most of the participants in the study were satisfied with the Tai Chi training course. Compared with the full form of Tai Chi practice, this short form Yang-style Tai Chi reduced the complexity and time required; hence participants could learn to practice within a relatively short period of time and kept a relatively good maintenance during the whole trial (35). Also, all the adverse effect reported were mild without affecting Tai Chi training, which suggested that Tai Chi was an acceptable and safe mind-body exercise for migraine patients.

Our study findings are in line with the effectiveness of nonpharmacological treatments like exercise (36, 37), acupuncture (28, 38), and other behavioral interventions (39) on the prophylaxis of episodic migraine. As a typical traditional Chinese medicine treatment, acupuncture showed a standardized mean difference (SMD) of 0.56 (95% CI: −0.65 to −0.48) reduction of headache frequency compared with no acupuncture (40). For those behavioral headache interventions with an aerobic exercise component, the headache frequency was reduced by 0.76 SMD (95% CI: 0.32–1.2) among six studies (41). A study conducted in 91 Swedes with episodic migraine showed that participants who conducted 40-minute aerobic exercise with three times per week for 3 months could averagely reduce 0.93 (95% CI: 0.31–1.54) attacks per month, it was comparable to the topiramate and relaxation training in the same treatment duration (37). Multiple levels of evidence support a role of aerobic exercise in migraine prevention and treatment: exertion reduces pain intensity, frequency, duration of attacks, and medication use; moreover, lower cardiovascular fitness levels increase the lifetime risk of developing migraine (42). Tai Chi training in our study showed stronger effect than those previous studies. On average, 3.6 migraine days per month reduction after the 12-week training was observed. We certainly would not ignore the placebo effect due to the waiting list control design, but we still believe that such large effect size indicates that Tai Chi has the potential to be “at least non-inferior” comparing conventional treatments for migraine prophylaxis. We consider it as reasonable inference because Tai Chi has shown its significant health benefits on many mental and neurological disorders (22, 24, 43). Of course, this clinical efficacy should be further carefully examined by comparing with regular pharmacological prophylaxis with enough observation periods in future studies.

We did not find significant differences regarding intensity and duration of headache between groups in the study. While in the Tai Chi group, the significant headache alleviation was observed within group when compared the data before and after the intervention. A recent review suggested that, mind–body interventions, including Tai Chi, had a positive effect on migraine and tension headaches (44). A cross-sectional study in German showed that frequent headache, severe impact of headache on daily life, and depressive symptoms were associated with more frequent analgesic use in both men and women, for women, physical inactivity was associated with higher frequency of analgesic use adjusted for sociodemographic and headache-related variables (45). Another systematic review and meta-analysis of 1,012 participants from 15 studies that assessed 6–20 weeks Tai Chi training for chronic pain, including headache, demonstrated that Tai Chi was associated with a significant reduction in headache (SMD: −1.85, −2.73 to −0.97). Tai Chi has a therapeutic value in the treatment of tension-type headache because of the mind-body interaction and relaxation effects (23, 46). It is our opinion that if the between-group differences are too small to discover in a sample of this size in our study, except the relative insufficient study power for testing headache alleviation, other factors than effect might be just as important to consider, such as participants' beliefs about Tai Chi, individual's bodily function, and the duration of the intervention, etc.

What biological and psychological mechanisms underlie the efficacy of Tai Chi as a prophylaxis treatment for migraine? Currently it is far from definitive. Some experts have deduced the mechanisms of aerobic exercise for the reducing of migraine burden and summarized the models of change processes (19). In general, the biological (neuroinflammatory, neurovascular, neurolimbic, neuroendocrine) and psychological (social-cognitive, social support, locus of control, mood state, tress, depression, anxiety) pathways operating independently, synergistically, or perhaps antagonistically, in the link between aerobic exercise and migraine improvement (17, 19, 36, 37, 47). Tai Chi is also an aerobic exercise, we believe that the mechanism of Tai Chi on the migraine prophylaxis would at least partially similar as other aerobic exercises. Studies showed that Tai Chi could improve white matter network (48), enhance cerebrovascular blood flow and reactivity (49), boost immunity (50), and avoid migraine trigger factors (25) such as stress, fatigue, and sleep quality. Moreover, Tai Chi involves movement of the whole body in fluidity and harmony, which requires concentration and mindfulness meditation. This may modulate multiple aspects of health, including mood, pain, and functions of the immune and peripheral autonomic nervous systems (51–53). Furthermore, low VO_2_ max is significantly associated with migraine (54), and the benefits of Tai Chi in improving VO_2_ max have been identified in literature (55), as well as in our previous study (56, 57). More studies with mechanism exploration in Tai Chi-migraine relationship are suggested.

There were some limitations in our study. Firstly, to consider the difficulty of administration and operation, double-blind study design was not used. Given that Tai Chi is behavior-based treatments, participants in the intervention groups might have higher expectations of the treatment results. Also, a waiting list control group was adopted. Thereby the placebo effects could not be ignored. Although the effect might be a little bit overestimated, we suggest that the evidence got from our study is valuable and could be considered as fundamental for future relevant studies, and an active control group is recommended. Secondly, measurements used in this study for migraine features (frequency, intensity, and duration) were subjective, which may have recall bias. But the migraine diary was a widely used tool with acceptable reliability and validity. It is unlikely to cause a significant impact on the results of outcomes. Thirdly, migraine types were not measured in our study, e.g., migraine with aura and migraine without aura. Since this is a pilot trial, we suggest that the relevant small sample size in each group might lead to the subgroup effect undetectable for migraine with aura cases. Fourthly, the 12 weeks of intervention might not be sufficient to observe long-term effectiveness of Tai Chi training on certain health outcomes, thus, extended intervention duration is suggested for future studies. Fifthly, the real dropout rate was a little bit higher than the designed dropout rate, the study power could be lowered a little bit. Whereas, we believed that the evidence obtained by both ITT and per protocol analyses in this pilot trial still has significant reference for future studies. Finally, compared with other studies adopting nonpharmacological prophylaxis, the participants in our study were relatively older (58, 59); and the sample size was relatively small. Although the study has acceptable power, we cannot exclude the notion that the differences of effectiveness on headache alleviation between groups exist. More studies with a larger sample size and a longer intervention period are needed to further evaluate the clinical efficacy of the Tai Chi training on the reduction of migraine burden. Despite these limitations, we believe that Tai Chi would be an easily adopted mind-body exercise with significant psychosocial and biophysiological benefits on the prevention of migraine attack.

## Conclusion

In conclusion, our study indicated that Tai Chi was an effective mind-body exercise in preventing migraine attack. Tai Chi can be well self-administered after a training period; it has the potential to empower migraineurs for their self-management of migraine. Evidence from our study provides a perspective that Tai Chi could be incorporated in integrative medicine that prompts physicians, healthcare providers, and healthcare policy makers to consider its efficacy in the whole management process. Future studies can be implemented to further examine any Tai Chi-migraine relief dose response exists, and whether Tai Chi can be synergistic with other behavioral or pharmacologic treatments. Furthermore, more advanced Tai Chi modality with mechanism-based exploration including neurovascular and neuroimaging examination, inflammatory factors and others would be adopted in migraine prevention with promising results.

## Data availability statement

The original contributions presented in the study are included in the article/Supplementary material, further inquiries can be directed to the corresponding author.

## Ethics statement

The studies involving human participants were reviewed and approved by the Human Subjects Ethics Sub-committee of the Hong Kong Polytechnic University (reference no.: HSEARS20160329002). The patients/participants provided their written informed consent to participate in this study.

## Author contributions

YJX conceived, designed the study, provided administrative, technical, or material support to the study, collected, analyzed the data, and drafted the manuscript. YJX, LT, SH, JQ, YG, DZ, TM, LS, HW, Z-ML, CH, LY, and AL made critical revisions of the manuscript. All authors read and approved the manuscript.
