# Supplementary material for: Efficacy and feasibility of a 12-week Tai Chi training for the prophylaxis of episodic migraine in Hong Kong Chinese women: A randomized controlled trial
Source: Front Public Health. 2022 Dec 13;10:1000594. doi: 10.3389/fpubh.2022.1000594 (PMC9792997; doi:10.3389/fpubh.2022.1000594)
Supplement: Supplementary file 1 [file Data_Sheet_1.docx]

**Supplementary material: Protocol of 12-week modified short form Yang-style Tai Chi training**

| 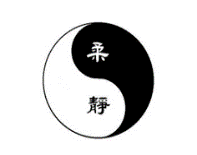 | **柔靜太極拳硏藝社**  **The Gentle and Tranquil Tai Chi Chuan Association** |
| --- | --- |

**楊家精簡貫串太極拳套路-三十三式**

**A modified 33-short form Yang-style Tai Chi Chuan**

**Format of each instructor-led lesson (1 hour/lesson, 3 lessons/week):**

1. Warm–up stretching (10mins)
2. Tai Chi Chuan practice (45mins)
3. Cool-down stretching (5mins)

**Content of Tai Chi Chuan practice (in total 36 lessons):**

The 33 forms of Yang-style Tai Chi Chuan will be taught in the first 15 lessons. The contents of lessons 1 to 15 are listed in the table below. Starting from lesson 16, participants will practice the whole set of 33-form Tai Chi Chuan for 3 times in each lesson. The time needed for completion of one whole set will be 15 mins. Two parallel Tai Chi classes were arranged for the participants. Class A was scheduled on Monday, Wednesday, and Friday; class B was set on Tuesday, Thursday, and Saturday.

| **Lesson** | **式 (Form)** |
| --- | --- |
| 1 | 1) 起勢 (Opening Form) |
| 2 | 2) 攬雀尾 (Grasp the Peacock’s Tail) |
| 3 | 3) 單鞭 (Single Whip) |
| 4 | 4) 玉女穿梭(4個) (Fair Lady Works Shuttles, 4 times); 5) 左掤 (Left Ward Off) |
| 5 | 6) 野馬分鬃(3個) (Part the Wild Horse’s Mane, 3 times); 7) 雲手(5次) (Cloud Hands, 5 times) |
| 6 | 8) 單鞭下勢　(Descending Single Whip);  9) 左右金雞獨立 (Golden Rooster Stands on One Leg, Left and Right) |
| 7 | 10) 左右倒攆猴 (Step Back to Repulse the Monkey, Left and Right); 11) 斜飛勢 (Diagonal Flying) |
| 8 | 12) 提手上勢 (Lift Hand); 13) 白鶴亮翅 (White Crane Spreads Its Wings);  14) 左右摟膝拗步(3次) (Brush Knee and Twist Step , Left and Right, 3 times) |
| 9 | 15) 左右分腳 (Toe Kick, Left and Right); 16) 轉身蹬腳 (Turn Body, Heel Kick);  17) 左右摟膝拗步(2次) (Brush Knee and Twist Step , Left and Right, twice) |
| 10 | 18) 左右打虎 (Hit the Tiger Left and Right); 19) 回身右蹬腳 (Turn Body and Right Heel Kick) |
| 11 | 20) 雙峰貫耳 (Strike to Ears with Both Fists); 21) 左蹬腳 (Left Heel Kick);  22) 轉身右蹬腳 (Turn Body and Right Heel Kick) |
| 12 | 23) 進步搬攔捶 (Step Forward, Move, Parry and Punch);  24) 上步攬雀尾 (Step Forward, Grasp the Peacock’s Tail) |
| 13 | 25) 單鞭下勢(Descending Single Whip); 26) 上步七星(Step Up to Seven Stars);  27) 退步跨虎 (Step Back to Ride the Tiger) |
| 14 | 28) 轉身雙擺蓮 (Turn Body, Lotus Kick); 29) 彎弓射虎 (Draw the Bow and Shoot the Tiger);  30) 進步搬攔捶 (Step Forward, Move, Parry and Punch) |
| 15 | 31) 如封似閉 (Apparent Close Up); 32) 十字手 (Cross Hands);  33) 收勢 (Closing Form) |
